# Attitudes, beliefs, and practices among Swiss chiropractors regarding medication prescribing for musculoskeletal conditions: a national Q-methodology study

**DOI:** 10.1186/s12998-020-00341-6

**Published:** 2020-10-20

**Authors:** Peter C. Emary, Mark Oremus, Taco A. W. Houweling, Martin Wangler, Noori Akhtar-Danesh

**Affiliations:** 1 Department of Health Research Methods, Evidence and Impact, McMaster University, Hamilton, ON Canada; 2 Chiropractic Department, D’Youville College, Buffalo, NY USA; 3 Private Practice, 1145 Concession Road, Cambridge, Ontario, N3H 4L5 Canada; 4 School of Public Health and Health Systems, University of Waterloo, Waterloo, ON Canada; 5 Private Practice, Faubourg de l’Hôpital, Neuchâtel, Switzerland; 6 Private Practice, Bern, Switzerland; 7 School of Nursing, McMaster University, Hamilton, ON Canada

**Keywords:** Chiropractic, Attitudes, Beliefs, Drug prescription, Switzerland, Q-methodology

## Abstract

**Background:**

Swiss chiropractors have been licensed since 1995 to prescribe from a limited formulary of medications for treating musculoskeletal (MSK) conditions. In January 2018, this formulary was expanded to include additional muscle relaxant, analgesic, and anti-inflammatory medications. Internationally, controversy remains over whether or not medication prescribing should be pursued within the chiropractic profession.

**Objective:**

The purpose of this study was to assess Swiss chiropractors’ attitudes, beliefs, and practices regarding their existing medication prescription privileges. This information will provide new insights on the topic and help inform research and policy discussions about expanding chiropractic prescription rights in other jurisdictions.

**Methods:**

A 13-item questionnaire and Q-methodology approach were used to conduct the assessment. Recruitment was conducted by e-mail between December 2019 and February 2020, and all members of the Swiss Chiropractic Association were eligible to participate. Data were analyzed using by-person factor analysis and descriptive statistics.

**Results:**

In total, 187 Swiss chiropractors participated in this study (65.4% response rate). Respondents reported prescribing analgesics, anti-inflammatories, and muscle relaxants to a median of 5, 5, and 0% of patients, respectively. Forty-two percent of respondents expressed interest in further expanding the range of current medications available to Swiss chiropractors for treating MSK conditions. Only 15% expressed interest in expanding this range to include medications for treating non-MSK conditions. In the Q-methodology analysis, four salient viewpoints/groups regarding medication prescribing emerged: *prescribers*, *non-prescribers*, *collaborators*, and *integrators*. All except *non-prescribers* thought medication prescription privileges were advantageous for the chiropractic profession in Switzerland. There was also strong consensus among all four groups that medication prescribing should not replace manual therapy in chiropractic practice.

**Conclusion:**

This was the first national survey on attitudes toward prescribing medications among Swiss chiropractors since the year 2000, and the first using Q-methodology. With this approach, four unique groups of chiropractic prescribers were identified. Even with diversity among clinicians, the findings of this study showed general support for, along with conservative use of, prescribing privileges within the Swiss chiropractic profession. Studies in jurisdictions outside of Switzerland are needed to assess whether chiropractors are interested in expanding their scopes of practice to include similar prescribing privileges.

## Introduction

The right to prescribe medications is a controversial topic within the chiropractic profession [1, 2]. At present, only nine countries, including Switzerland, Liechtenstein, Guatemala, Panama, United Arab Emirates, India, Iran, Namibia, and the United States (New Mexico and Oklahoma, only) allow for such privileges [3]. However, evidence suggests that chiropractors who have medication prescription rights perceive these privileges as an advantage for the profession [4, 5]. Moreover, if granted limited prescriptive authority (i.e., limited to prescribing medications for treating spine-related and other musculoskeletal [MSK] conditions), chiropractic clinicians could have a positive influence on public health [2]. This is because, with such privileges, chiropractors would be in a position to counsel patients with MSK pain against overusing or over-relying on medications commonly prescribed to treat their condition. In fact, under federal law [6], chiropractors in Switzerland can prescribe from a limited formulary of muscle relaxants, non-steroidal anti-inflammatory drugs (NSAIDs), and analgesics (Table 1). In addition, studies have shown that Swiss chiropractors exercise judicious use of prescribing in clinical practice [5, 7, 8]. However, the clinical circumstances under which Swiss chiropractors prescribe medications are largely unknown. Rates of prescribing medications for MSK conditions across the Swiss chiropractic profession have also not been rigorously assessed.

**Table 1:** Chiropractic formulary in Switzerland ^a^

Therapeutic group	Active ingredient
Antipyretic analgesics	Paracetamol, Acetylsalicylic acid
	Metamizole, Lysini acetylsalicylicum
Myotonolytics (administered by oral solution only)	Tolperisone (Mydocalm®)
	Tizanidine (Sirdalud®)
	Baclofen (Lioresal®)
Gastroenterologics (only proton pump inhibitors)	Esomeprazole, omeprazole
	Lansoprazole, pantoprazole
	Rabeprazole, dexlansoprazole
Minerals	Magnesium (e.g., Magnesiocard®, Diasporal®)
Simple vitamins	Calcitriol, Cholecalciferolum, Vitamin D (e.g., Renatriol®, Rocaltrol®, Vitamin D3 Streuli®)
Simple anti-inflammatory agents	Examples: Ibuprofen, Naproxen, Dicolfenac, Piroxicam, Lornoxicam, Nimesulidum, Flurbiprofenum, Indometacinum, Cexketoprofenum, Etodolacum, Acidum mefenacidum, Meloxicamum, Dexibuprofenum, Tenoxicamum, Acemetacinum, ...
Combined anti-inflammatory agents without corticosteroids (only in combination with proton pump inhibitors)	Naproxen + Esomeprazole (Vimovo®)
Other	Chondroitin sulfate (Condrosulf®)
Neural therapeutics	Lidocaine, Procain

^a^ Information provided by the Cantonal Pharmacist Office, Bern, Switzerland

The purpose of this study was to assess Swiss chiropractors’ current attitudes toward, the frequency of, and indications for, medication prescribing for MSK conditions in clinical practice. Based on previous literature [1, 2, 5, 9–12], this study also aimed to explore Swiss chiropractors’ beliefs toward their current pharmacology training, as well as their interest in expanding the current Swiss prescribing formulary to include additional medications for MSK and non-MSK conditions.

The results of this study are important because they can be used by clinicians, educators, decision-makers, and health policy-makers to inform future directions and research regarding prescribing practices for chiropractors in Switzerland, and possibly in other jurisdictions. For instance, reports indicate that a growing number of chiropractors from countries outside Switzerland are also interested in expanding their scopes of practice to include similar prescribing privileges [10–17]. However, debate continues over the standards of pharmacology training for chiropractors internationally, as well as the extent to which medication prescribing rights should be expanded within the profession [1, 2, 9–12].

## Methods

### Study design

**Fig. 1:**
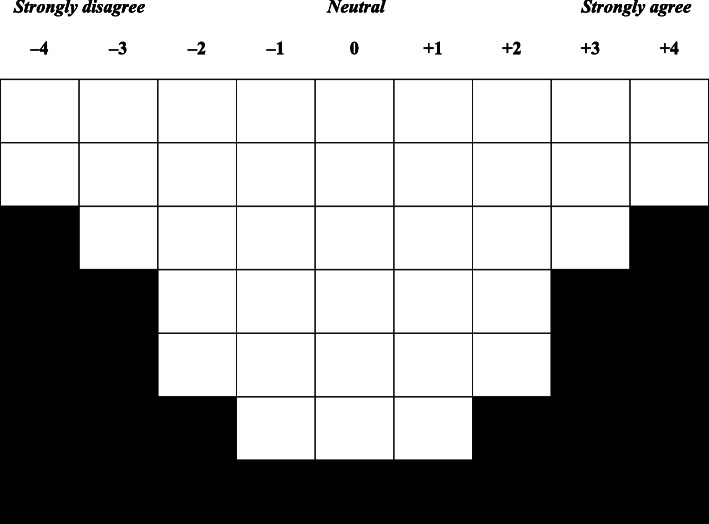
Q-sort table used for rank-ordering Q-sample statements. This Q-sort table has 38 spaces (or ranking positions), anchored from − 4 to + 4, and is designed to be used with a set of 38 statements

We conducted a cross-sectional study of chiropractors across Switzerland and employed a Q-methodological approach to identify major viewpoints held about medication prescribing for MSK conditions among study participants [18, 19]. The study was conducted in two phases. First, we developed a study instrument, or *Q-sort table* [18, 19] (Fig. 1), and paired it with a demographic questionnaire [20]. Second, these two instruments were employed to collect data. We implemented the Q-sort table using a freely downloadable app (i.e., Lloyd’s Q Sort Tool [www.nowhereroad.com/qsort/]), and administered the demographic questionnaire through SurveyMonkey (www.surveymonkey.com). The instruments were developed in English because the target population was able to communicate in this language.

### Q-methodology

Our study marks the first use of Q-methodology for research within the chiropractic profession. Our rationale for using Q-methodology was that it has several important advantages over traditional survey methods. First, this methodology combines quantitative and qualitative techniques [18, 19], and thereby produces more complete or holistic data regarding participant viewpoints on a given topic, particularly when compared to quantitative (e.g., Likert-scale) items on a survey [19]. Q-methodology has also been shown to be useful in eliciting subjective viewpoints [18, 19]. This is particularly advantageous in topic areas, such as prescribing rights in chiropractic, where more conventional methods (i.e., closed-ended quantitative surveys) have not elucidated a clear definition or consensus on the subject matter [1, 2, 18, 19]. A further advantage of Q-methodology is that a low response rate does not bias a study’s results. This is because the aim of Q-methodology is to identify a typology of ideas, not the frequency and distribution of responses to questions [18, 21]. Therefore, for statistical purposes, a sample size of between 40 and 60 participants is usually sufficient for a Q-methodology survey [18, 22].

### Phase 1: instrument development

#### Concourse

The methods for instrument development and administration have been detailed in our published study protocol [20]. Briefly, in November 2019, we developed a *concourse* using 271 statements elicited from a purposive sample of 27 of 30 Swiss chiropractors (90% response proportion). A concourse is a list of comprehensive descriptive statements on the topic of interest [18, 19]; these statements are used to build the final set of items, or *Q-sample*, that are included in the Q-methodology survey (see below). We added 54 additional statements to the chiropractors’ initial list of 271; these statements were informed by the literature [1, 2, 4, 5, 8, 10–12] and our content expertise. Using structured and inductive thematic analysis methods [18–20], the statements were then categorized into eight major themes and 13 sub-themes. The eight major themes included attitudes supporting medication prescribing (69 statements), attitudes opposed to medication prescribing (76 statements), a recognition of the need for continuing education in pharmacology (27 statements), beliefs regarding current pharmacology training (49 statements), clinical indications used when prescribing (25 statements), conservative attitudes/practices toward prescribing (34 statements), collaborating with general practitioners or pharmacists when prescribing (15 statements), and the scope of prescribing in chiropractic practice (30 statements).

#### Q-sample

The statements within each of the major themes of the concourse were reviewed for similarities and differences by two investigators, and duplicate/redundant statements were removed. Through iterative discussions among the investigators, as described elsewhere [20], we consolidated all of the statements into a final list of 38 statements (i.e., the Q-sample). This list broadly represented the key ideas from all of the conceptual and emerging themes about Swiss chiropractors’ attitudes and beliefs toward prescribing medications for MSK conditions. We then developed and pilot tested the Q-sort table to match the total number of statements in the Q-sample (see Fig. 1) [20]. The 38 statements used in the final Q-sample are presented in Additional file 1. We also developed a 13-item demographic questionnaire to accompany the Q-sample [20].

### Phase 2: data collection

To recruit participants for this study, the administrative offices of the Swiss Chiropractic Association (ChiroSuisse) sent an e-mail information letter to all ChiroSuisse members (*n* = 286) in December 2019 [23]. The letter contained links to the Q-sort table and demographic questionnaire. As per the Dillman method [24], the letter was preceded by a notice published in the association’s December 2019 newsletter, sent one week prior to the start of the study. The initial e-mail letter was followed by three reminder notifications, sent between December 2019 and January 2020. ChiroSuisse also sent a final reminder in February 2020 asking non-respondents to complete the demographic questionnaire.

Both the Q-sort table and demographic questionnaire were completed by each participant independently. For the Q-sort table, participants were asked to read the list of 38 statements and place each statement into an empty cell corresponding to the amount of agreement they had with each statement. Any statement placed under a negative number indicated disagreement (or less agreement), and any statement placed under a positive number indicated agreement. Participants were instructed to sort the statements in this manner until they filled all cells in the Q-sort table [18, 19]. Participants were then asked three open-ended questions about why they sorted the items the way they did, as well as a 9-point Likert-style question, ranging from − 4 (strongly disagree) to + 4 (strongly agree), about their level of agreement with the following statement, “*I think that medication prescription privileges are an advantage for the chiropractic profession in Switzerland*.”

### Data analysis

We generated frequencies for all collected data and compared demographic characteristics of Q-sort respondents with non-respondents using chi-square and t-tests (or Fisher exact and Wilcoxon-Mann-Whitney tests as appropriate). All data and comparative analyses were performed using SAS® v9.4 (SAS Institute Inc., Cary, North Carolina). The statistical significance level (*α*) for quantitative analyses was 5%.

To implement the Q-methodology, a by-person factor analysis [18, 19] of the completed Q-sorts was used to investigate salient viewpoints, as well as shared viewpoints, among participants. Participants’ demographic information and written comments were also triangulated to their completed Q-sort data to aid in factor interpretation [19, 20]. Relationships between factors and demographic variables were explored using chi-square and one-way analysis of variance (ANOVA) tests (or Fisher exact and Kruskal-Wallis tests as appropriate). Pertinent comments provided by significantly loading participants were then presented alongside factor scores and distinguishing statements.

### Factor extraction and rotation

We used the **qfactor** command in Stata [25] and employed iterated principal axis factor-extraction and varimax rotation procedures [18–20]. Following factor extraction and factor rotation, a weighted (or synthetic) Q-sort was produced for each rotated factor using a weighted averaging method to calculate the score for each statement for that factor [19, 21]. Each factor was then assigned a name that reflected the factor configuration. Names were assigned to each factor based on the factor’s *distinguishing* statements (i.e., statements that scored statistically significantly different on that factor compared to any other factor) [18, 19]. We used a medium Cohen’s effect size of 0.5 to identify distinguishing statements [20, 25, 26].

### Ethical considerations

Prior to data collection, ethics approval was obtained from the Hamilton Integrated Research Ethics Board at McMaster University (approval number 2019–7612). Local approval in Switzerland was also obtained from the Swiss Cantonal Ethics Commission (approval number 2019–00926). Chiropractors who completed either the Q-sort table or demographic questionnaire were assumed to have given implied consent to participate in the study.

## Results

In total, 187 of 286 ChiroSuisse members (65.4%) participated in the study. Of these, 185 completed the demographic questionnaire and 91 completed the Q-sort. Two of the 187 participants did not complete the demographic questionnaire, and two of the 91 Q-sorts were excluded because of missing data. This resulted in 185 questionnaires and 89 usable Q-sorts for data analysis. Comparisons of demographic characteristics between Q-sort respondents and non-respondents are provided in Additional files 2 and 3.

A summary of the demographic data and scope of practice characteristics for all study participants is displayed in Table 2. Regarding the frequency of medication prescribing by Swiss chiropractors, participants reported prescribing analgesics, NSAIDs, and muscle relaxants to a median of 5% (inter-quartile range [*IQR*] = 0 − 11%), 5% (*IQR* = 1 − 12%), and 0% (*IQR* = 0–3%) of patients, respectively. Of the 185 participants, 77 (41.6%) were also interested in expanding the range of current medications available to Swiss chiropractors for treating MSK conditions. However, only 28 (15.1%) expressed interest in expanding this range to include medications for treating non-MSK conditions (e.g., antibiotics, anti-hypertensives, anti-depressants, etc.).

**Table 2:** Summary of demographic and scope of practice characteristics of study participants (*n* = 185)

Variable	***n*** (%) ^**a**^
Age (years): mean (*SD*)	50.3 (11.3)
Gender	
• Female	70 (37.8)
• Male	115 (62.2)
Chiropractic school of graduation	
• Canadian Memorial Chiropractic College	40 (21.6)
• Western States Chiropractic College	31 (16.8)
• Palmer College of Chiropractic West	22 (11.9)
• Palmer College of Chiropractic	20 (10.8)
• Southern California University of Health Sciences	19 (10.3)
• Other ^b^	53 (28.6)
Region of practice	
• Swiss-German	127 (68.7)
• Swiss-French	47 (25.4)
• Swiss-Italian	11 (5.9)
Years in practice: mean (*SD*)	22.5 (10.9)
Postgraduate university degree (e.g., MSc, PhD)	31 (16.8)
Work in multidisciplinary practice/hospital setting	53 (28.7)
Collaborate with GP and/or specialists on a daily basis	133 (71.9)
Frequency of medication prescribing in clinical practice: median (*IQR*)	
• Analgesics, % of patients	5 (0–11)
• NSAIDs, % of patients	5 (1–12)
• Muscle relaxants, % of patients	0 (0–3)
Interest in expanding range of current medications available to prescribe for MSK conditions (e.g., opioids, corticosteroids)	77 (41.6)
Interest in expanding range of current medications available to prescribe for non-MSK conditions (e.g., antibiotics, anti-hypertensives, anti-depressants, etc.)	28 (15.1)

*GP* general practitioner, *IQR* inter-quartile range, *MSK* musculoskeletal, *NSAIDs* non-steroidal anti-inflammatory drugs, *SD* standard deviation

^a^ Values are expressed as the number (%) unless otherwise noted

^b^ Included graduates from the National University of Health Sciences (*n* = 12), Northwestern College of Chiropractic (*n* = 9), University of Zürich (*n* = 9), Institut Franco-Européen de Chiropratique (*n* = 8), AECC University College (*n* = 5), New York Chiropractic College (*n* = 4), Logan College of Chiropractic (*n* = 3), Cleveland Chiropractic College (*n* = 1), Texas Chiropractic College (*n* = 1), and the Université de Québec à Trois Riviéres (*n* = 1)

### Factors

Using a by-person factor analysis of the Q-sort data, four factors (i.e., salient viewpoints) emerged regarding Swiss chiropractors’ attitudes and beliefs toward prescribing medications for MSK conditions. These four factors included 76 (85.4%) of the 89 Q-sort participants. The remaining 13 participants who did not load significantly (*p* < 0.05) on any of these factors were excluded from further comparative analyses among the four factors [18]. The factors were named as follows: (i) *prescribers*, (ii) *non-prescribers*, (iii) *collaborators*, and (iv) *integrators*.

#### Factor 1: prescribers. “Prescription rights are an important tool in MSK care”

This factor was represented by 38 significantly loading Q-sorts (participants). The chiropractors in this group had an average of 20.5 (standard deviation [*SD*] = 9.7) years of clinical experience, eight worked in a multidisciplinary/hospital-based setting, and 25/37^1^ (67.6%) collaborated with other medical professionals (e.g., general practitioner and/or specialist) on a daily basis. The age range was 27–64 years.

*Prescribers* reflected a generally favourable attitude toward prescribing medications in chiropractic practice (Table 3). They strongly (+ 4) felt that, as MSK health specialists, chiropractors should have access to a variety of treatment options including medication. In their Q-sort comments, several indicated that their existing prescription rights were “an important tool” and were “in line with [evidence-based] guidelines.” Others suggested that chiropractors with prescribing privileges could improve the efficiency of MSK care and “lower health costs,” a statement supported by some published evidence [8]. *Prescribers* also strongly (+ 4) believed that prescribing painkillers and NSAIDs was a useful addition to chiropractic practice, particularly for treating patients who were in severe pain (statement 35). As summarized by one respondent,

*[With prescription rights], I can better control the healing of the patient, reassure him, prescribe exercises, and finish the case with less costs than the medical doctor. P 122*

*Prescribers* strongly disagreed (− 4) with the statement that “medication prescription should only be performed by general practitioners or pharmacists” (statement 10). In their Q-sort comments, a common theme among many of these respondents was that chiropractors should be allowed to prescribe medications. For example,

*Why should it be forbidden [for] chiropractors to make use of a treatment tool that can be beneficial to the patient? This makes no sense. P 132*

Some also felt that such privileges should not be denied by the chiropractic profession simply for ideological reasons. As stated by the following respondent,

*Medication, “natural” or “synthetic,” should be an option in the treatment of MSK pain and [should] not be automatically excluded because of purely dogmatic rhetoric. P 23*

Some *prescribers* suggested that the chiropractic profession should move on from its historical past to become a more modern and integrated healthcare profession. For instance,

*We are in a time where we drive cars and airplanes, not buggies and horse chariots. We have to evolve to [be able to provide] the best care possible and [deliver it in] the most efficient way. P 122*

Overall, *prescribers* strongly agreed (+ 3.5) that medication prescription privileges were an advantage for the chiropractic profession in Switzerland (see Table 3).

**Table 3:** Distinguishing statements, factor scores, and summary statement score for *prescribers* (Factor 1) ^a^

Statement		Factor 1	Factor 2	Factor 3	Factor 4
37	I think that as MSK health specialists, chiropractors should have access to a variety of treatment options including medication.	**4**	–1	–1	−2
35	I feel that prescribing painkillers and NSAIDs is a useful addition to chiropractic, particularly for patients who are in severe pain.	**4**	0	1	0
32	I prescribe medication only during acute and sub-acute episodes of pain, not for long-term use.	**3**	−1	0	0
25	I think medication prescription rights for chiropractors can streamline care, helping patients with MSK complaints to avoid unnecessary visits to their medical doctor.	**2**	0	–1	0
33	I think prescribing medication is a good adjunctive to our treatment in some instances to speed up recovery.	**1**	–1	–1	–1
36	I only prescribe pain medication when I think it would be useful, not every time the patient asks.	**1**	–1	–2	–1
21	I think that our current medication prescription privileges are in line with current evidence-based practice.	**1**	0	–2	0
9	I think that being allowed to prescribe increases our credibility among patients.	**0**	–2	2	3
34	I think that instead of prescribing, chiropractors should collaborate with the patient’s medical doctor for the prescription of medications.	**–1**	2	1	–2
15	I think our prescription rights in Switzerland should be open at least to level 2 analgesics (i.e., codeine, tramadol).	**–1**	−3	−3	1
6	I do not like to prescribe because it could interfere with other medical prescriptions (i.e., double prescription).	**−1**	1	4	−3
3	I think medication prescriptions are a burden because they bring added professional responsibility to the chiropractor.	**−2**	0	1	−4
19	I think a chiropractor prescribing medications is like a medical doctor doing manipulations, let us each focus on what we do best.	**−3**	4	1	−2
5	I think the use of medication for back pain should be discouraged and Swiss chiropractors should lead the way.	**−3**	3	0	−1
26	I believe it should be part of the definition of Chiropractic that we assist the body in self-healing WITHOUT the use of drugs or surgery.	**−3**	4	−1	−3
10	I think medication prescription should only be performed by general practitioners or pharmacists.	**−4**	2	0	−3
**Summary statement:**		**Median (*IQR*) summary statement score:**			
*I think that medication prescription privileges are an advantage for the chiropractic profession in Switzerland.*		3.5 (2 to 4)			

*IQR* inter-quartile range, *MSK* musculoskeletal, *NSAIDs* non-steroidal anti-inflammatory drugs

^a^ Factor and summary statement scores range from − 4 to + 4. Negative scores indicate disagreement (or less agreement)

#### Factor 2: non-prescribers. “We should be providing the alternative”

This factor was represented by 24 participants. *Non-prescribers* had an average of 22.6 (*SD* = 11.7) years of clinical experience, eight worked in a multidisciplinary/hospital-based setting, and 13/23^2^ (56.5%) collaborated with other medical professionals including general practitioners and/or specialists on a daily basis. The age range was 30–79 years.

Contrary to *prescribers*, *non-prescribers* were generally opposed to medication prescribing in chiropractic practice (Table 4). They felt strongly (+ 4) that “a chiropractor prescribing medications was like a medical doctor doing manipulations,” and that each health care professional “should focus on what they do best.” In addition, *non-prescribers* strongly (+ 4) believed that it should be part of the definition of the chiropractic profession that chiropractors “assist the body in self-healing WITHOUT the use of drugs or surgery,” a sentiment reflective of the profession’s international history [1]. This is also reflected in the comments given by the following respondent:

*I don’t think the chiropractic profession should be blending [its scope of practice] with the medical profession to such a degree. We need to stay separate and apart and not give up our roots! P 128*

Some respondents indicated that patients often visit a chiropractor for pain management because medications and other therapies have been ineffective. For instance,

*One very common reason why patients seek care in my clinic is because they have already tried the medications, injections, cortisone shots, etc. [and these] have not given them the results they [had] hoped for. P 103*

*Non-prescribers* also strongly disagreed (− 4) that adding new drug classes (opioids) to their prescription rights would be useful (statement 2). This particular respondent stated that,

*Too many other health care providers prescribe medication (including opioids). We should be the ones providing the alternative. P 14*

In fact, interest in expanding the current formulary to include opioids and corticosteroids, as measured with the demographic questionnaire, was significantly lower among *non-prescribers* compared with *prescribers*, *collaborators*, and *integrators* (0% vs. 54, 44, and 60%, respectively; *p* < 0.001). Moreover, *non-prescribers* were the only group out of the four to disagree (− 1.5) with the statement that, “*medication prescription privileges are an advantage for the chiropractic profession in Switzerland*” (see Table 4).

**Table 4:** Distinguishing statements, factor scores, and summary statement score for *non-prescribers* (Factor 2) ^a^

Statement		Factor 1	Factor 2	Factor 3	Factor 4
19	I think a chiropractor prescribing medications is like a medical doctor doing manipulations, let us each focus on what we do best.	− 3	**4**	1	− 2
26	I believe it should be part of the definition of Chiropractic that we assist the body in self-healing WITHOUT the use of drugs or surgery.	− 3	**4**	− 1	− 3
12	I personally take very little to no medication on a yearly basis and think we should encourage this same approach with our patients.	0	**3**	2	0
5	I think the use of medication for back pain should be discouraged and Swiss chiropractors should lead the way.	−3	**3**	0	−1
20	I believe patients choose to see a chiropractor because his/her therapy is drugless.	0	**3**	2	1
34	I think that instead of prescribing, chiropractors should collaborate with the patient’s medical doctor for the prescription of medications.	−1	**2**	1	− 2
27	I think that ice and painkillers, which are available without a prescription, are sufficient for our needs.	− 2	**2**	0	− 2
10	I think medication prescription should only be performed by general practitioners or pharmacists.	− 4	**2**	0	− 3
16	I feel that prescribing medication is useful in helping patients who cannot sleep because of pain.	1	**1**	2	3
22	I prescribe medication in extremely acute cases where absolutely no range of motion can be achieved and pain levels are too high.	3	**1**	− 2	2
6	I do not like to prescribe because it could interfere with other medical prescriptions (i.e., double prescription).	− 1	**1**	4	− 3
23	I think a review of new medication relevant to chiropractic practice should be organized for the profession every 2–5 years.	2	**0**	3	3
3	I think medication prescriptions are a burden because they bring added professional responsibility to the chiropractor.	− 2	**0**	1	− 4
17	I believe that chiropractors should get more continuing education (CE) about medications and side effects.	3	**0**	3	2
14	I believe that continuous education concerning medication prescription should be mandatory.	2	**− 1**	4	2
9	I think that being allowed to prescribe increases our credibility among patients.	0	**− 2**	2	3
4	I believe that medication prescription rights give us better credibility among our medical colleagues.	0	**− 2**	1	4
2	I think adding new drug classes (opioids) to our prescription rights would be useful.	−2	**−4**	− 2	− 1
**Summary statement:**		**Median (*IQR*) summary statement score:**			
*I think that medication prescription privileges are an advantage for the chiropractic profession in Switzerland.*					
		−1.5 (− 3 to −0.5) ^b^			

*IQR* inter-quartile range

^a^ Factor and summary statement scores range from − 4 to + 4. Negative scores indicate disagreement (or less agreement)

^b^ Pairwise comparisons indicated significantly different summary statement scores between Factor 2 and Factors 1, 3, and 4 (*p* < 0.001, *p* = 0.003, and *p* = 0.005, respectively)

#### Factor 3: collaborators. “We had only one course in toxicology”

This factor was represented by nine participants. *Collaborators* had an average of 21.7 (*SD* = 6) years of clinical experience, two worked in a multidisciplinary/hospital-based setting, and seven (77.8%) collaborated with other medical professionals on a daily basis. The age range was 41–56 years.

Similar to the *non-prescribers*, *collaborators* were generally opposed to medication prescribing in chiropractic practice (Table 5). However, for *collaborators*, this view mainly reflected a belief regarding the adequacy of their current pharmacology training rather than for reasons related to scope of practice. For instance, *collaborators* strongly (+ 4) believed that continuous education concerning medication prescribing should be mandatory (statement 14). They also strongly agreed (+ 4 and + 3, respectively) with the statements, “I do not like to prescribe because it could interfere with other medical prescriptions (i.e., double prescription),” and “chiropractors should get more continuing education (CE) about medications and side effects.” These concerns are further expressed by the following respondent:

*Medical doctors are trained in pharmacology, we had only one course in toxicology, not enough to take [on] that kind of responsibility. P 51*

*Collaborators* also strongly disagreed (− 3 and − 4, respectively) that their knowledge of, and chiropractic training for, prescribing medications to treat MSK or non-MSK conditions was sufficient (statements 1 and 8). As such, many of these chiropractors reflected the sentiment that medication prescribing should be co-managed with their medical colleagues, as elucidated by the following respondent:

*I personally have [a] very good rapport with local MDs [medical doctors] whom I call and ask … for drug support – which is very beneficial for our interprofessional rapport and also reduces many potential risks (like drug interaction, us not knowing what other drugs are used by our patients, etc.). P 8*

Overall, *collaborators* still agreed (+ 1) that medication prescription privileges were an advantage for the chiropractic profession in Switzerland, albeit to a lesser extent than did *prescribers* (*p* = 0.003) (see Table 5).

**Table 5:** Distinguishing statements, factor scores, and summary statement score for *collaborators* (Factor 3) ^a^

Statement		Factor 1	Factor 2	Factor 3	Factor 4
14	I believe that continuous education concerning medication prescription should be mandatory.	2	−1	**4**	2
6	I do not like to prescribe because it could interfere with other medical prescriptions (i.e., double prescription).	−1	1	**4**	−3
17	I believe that chiropractors should get more continuing education (CE) about medications and side effects.	3	0	**3**	2
28	I am concerned when prescribing medication that the patient may omit information from their medical history (e.g., Oh yes, I am taking Beta blockers, but that is none of your concern is it?...).	−1	1	**2**	−1
9	I think that being allowed to prescribe increases our credibility among patients.	0	− 2	**2**	3
12	I personally take very little to no medication on a yearly basis and think we should encourage this same approach with our patients.	0	3	**2**	0
3	I think medication prescriptions are a burden because they bring added professional responsibility to the chiropractor.	−2	0	**1**	−4
34	I think that instead of prescribing, chiropractors should collaborate with the patient’s medical doctor for the prescription of medications.	−1	2	**1**	−2
19	I think a chiropractor prescribing medications is like a medical doctor doing manipulations, let us each focus on what we do best.	− 3	4	**1**	− 2
27	I think that ice and painkillers, which are available without a prescription, are sufficient for our needs.	−2	2	**0**	−2
10	I think medication prescription should only be performed by general practitioners or pharmacists.	−4	2	**0**	− 3
7	The Apotheker/pharmacien (pharmacists) are for me the best people to contact with questions regarding medication.	1	2	**0**	2
5	I think the use of medication for back pain should be discouraged and Swiss chiropractors should lead the way.	− 3	3	**0**	− 1
38	I believe medications should be used conservatively in regards to patient management for MSK conditions.	2	2	**0**	− 2
26	I believe it should be part of the definition of Chiropractic that we assist the body in self-healing WITHOUT the use of drugs or surgery.	− 3	4	**−1**	− 3
22	I prescribe medication in extremely acute cases where absolutely no range of motion can be achieved and pain levels are too high.	3	1	**− 2**	2
21	I think that our current medication prescription privileges are in line with current evidence-based practice.	1	0	**− 2**	0
1	I feel my chiropractic training has adequately prepared me for prescribing medications to treat MSK conditions.	−1	− 2	**− 3**	0
8	I think my knowledge for prescribing medications for treating non-MSK conditions is sufficient.	−2	− 3	**−4**	2
**Summary statement:**		**Median (*IQR*) summary statement score:**			
*I think that medication prescription privileges are an advantage for the chiropractic profession in Switzerland.*					
		1 (1 to 2)			

*IQR* inter-quartile range, *MSK* musculoskeletal

^a^ Factor and summary statement scores range from − 4 to + 4. Negative scores indicate disagreement (or less agreement)

#### Factor 4: integrators. “It’s a necessary tool for the primary care practitioner”

This factor was represented by five participants. These chiropractors had an average of 24 (*SD* = 14.6) years of clinical experience, one worked in a multidisciplinary/hospital-based setting, and all five (100%) collaborated with medical professionals on a daily basis. The age range was 29–70 years.

Similar to *prescribers*, the *integrators* favoured medication prescribing in chiropractic practice (Table 6). In particular, *integrators* perceived prescription privileges as a distinct advantage (+ 4) for the chiropractic profession in Switzerland. They strongly (+ 4) believed that their medication prescription privileges allowed for better integration within the healthcare system (statement 11). In their Q-sort comments, some respondents also suggested that the responsibility of prescribing raised the standard of practice for chiropractors in Switzerland. For example, this respondent stated the following:

*I find that [because] we can give medication, it’s a privilege and it’s a big difference for our statute between us and the [chiropractic profession in] other countries. P 142*

*Integrators* also strongly agreed (+ 4) that medication prescription rights give them better credibility among their medical colleagues and their patients (statements 4 and 9, respectively). One respondent also commented on how these privileges are needed for chiropractors to have greater autonomy and cultural authority:

*…I believe that medication (excluding opioids) is a necessary tool to be able to fully assume the responsibility and independence of a primary care practitioner. P 65*

Furthermore, *integrators* strongly disagreed (− 4) with the statement that “medication prescriptions are a burden because they bring added professional responsibility to the chiropractor” (statement 3). One respondent (*P 2*) quipped that this “*is a statement for lazy chiropractors.*”

**Table 6:** Distinguishing statements. Factor scores, and summary statement score for *integrators* (Factor 4) ^a^

Statement		Factor 1	Factor 2	Factor 3	Factor 4
11	I feel our medication prescription privileges have allowed for better integration within the healthcare system.	0	−1	−1	**4**
4	I believe that medication prescription rights give us better credibility among our medical colleagues.	0	−2	1	**4**
9	I think that being allowed to prescribe increases our credibility among patients.	0	−2	2	**3**
16	I feel that prescribing medication is useful in helping patients who cannot sleep because of pain.	1	1	2	**3**
8	I think my knowledge for prescribing medications for treating non-MSK conditions is sufficient.	−2	−3	−4	**2**
15	I think our prescription rights in Switzerland should be open at least to level 2 analgesics (i.e., codeine, tramadol).	−1	−3	−3	**1**
31	I feel that in acute cases, pain medications can be used to alleviate the increased pain (i.e., normal side-effect during the first 24–48 h) due to the manipulation.	−1	− 2	−1	**1**
5	I think the use of medication for back pain should be discouraged and Swiss chiropractors should lead the way.	−3	3	0	**−1**
38	I believe medications should be used conservatively in regards to patient management for MSK conditions.	2	2	0	**−2**
34	I think that instead of prescribing, chiropractors should collaborate with the patient’s medical doctor for the prescription of medications.	−1	2	1	**−2**
19	I think a chiropractor prescribing medications is like a medical doctor doing manipulations, let us each focus on what we do best.	−3	4	1	**−2**
26	I believe it should be part of the definition of Chiropractic that we assist the body in self-healing WITHOUT the use of drugs or surgery.	−3	4	−1	**−3**
10	I think medication prescription should only be performed by general practitioners or pharmacists.	−4	2	0	**−3**
6	I do not like to prescribe because it could interfere with other medical prescriptions (i.e., double prescription).	−1	1	4	**−3**
3	I think medication prescriptions are a burden because they bring added professional responsibility to the chiropractor.	−2	0	1	**−4**
**Summary statement:**		**Median (*IQR*) summary statement score:**			
*I think that medication prescription privileges are an advantage for the chiropractic profession in Switzerland.*					
		4 (4 to 4) ^b^			

*IQR* inter-quartile range, *MSK* musculoskeletal

^a^ Factor and summary statement scores range from − 4 to + 4. Negative scores indicate disagreement (or less agreement)

^b^ Kruskal-Wallis test indicated significantly different summary statement scores among the four factors (*p* < 0.001). Pairwise comparisons revealed significantly different scores between Factor 4 and Factors 2 and 3 (*p* = 0.005 and *p* = 0.015, respectively), but a similar score to Factor 1 (*p* = 0.480)

When comparing factors, *integrators’* frequency of medication prescribing was significantly higher than that of their chiropractic colleagues. For instance, compared with *prescribers*, *non-prescribers* and *collaborators*, *integrators* reported prescribing analgesics to a median of 25% of patients (vs. 5, 0, and 1% of patients, respectively; *p* < 0.001), NSAIDs to a median of 25% of patients (vs. 5, 1, and 1% of patients, respectively; *p* < 0.001), and muscle relaxants to a median of 10% of patients (vs. 1, 0, and 0% of patients, respectively; *p* < 0.001).

There were no statistically significant relationships between the four factors and any of the remaining demographic variables, including age, sex, number of years in practice, chiropractic school of graduation, postgraduate university education, collaboration with other medical professionals, type of practice (e.g., solo, multidisciplinary, or hospital-based), and interest in expanding the range of current medications available to prescribe for non-MSK conditions.

### Consensus statements

*Consensus statements* are statements for which all participants in a Q-methodology study, regardless of factor, generally agree or disagree with to a similar extent [25]. In the current study, there was only one statement (number 18) whose factor scores were not significantly different between the four different factors: “I do not manipulate patients much anymore because prescribing medications is faster and easier.” Participants in all four groups strongly disagreed (− 4) with this statement. This was also reflected in several respondents’ comments, including those of *prescribers* and *integrators*. For example,

*I am not going to stop treat [ing] manually because of medication. P 32*

*Medical prescription is a part of our education at the University of Zürich and has to be allowed and also used! [However,] …our main work is and remains the manual therapy, which includes manipulation and should never be replaced by prescribing medications! P 123*

And,

*Whoever agrees with [statement] No. 18 should be kicked out of the profession! P 82*

## Discussion

This was the first national study of chiropractors in Switzerland on attitudes, beliefs, and practices regarding medication prescribing for MSK conditions conducted since the year 2000 [4]. It was the first such study to inquire about attitudes toward prescribing additional MSK (e.g., opioid) and non-MSK medications, as well as indications for prescribing, and the first to do so using Q-methodology [18, 19]. With this approach, we found four distinct viewpoints regarding medication prescribing among Swiss chiropractors, namely *prescribers*, *non-prescribers*, *collaborators*, and *integrators*.

In previously conducted quantitative surveys of Swiss chiropractors [4, 5], majorities (i.e., 72 and 82%) of respondents indicated that medication prescription privileges were an advantage for the chiropractic profession. These findings are supported by the current study because three of the four groups of ChiroSuisse members (i.e., *prescribers*, *collaborators*, and *integrators*) also agreed that the ability to prescribe from a limited formulary of medications was advantageous for the profession. However, these four identified groups also represented four distinct perspectives or viewpoints about medication prescribing for MSK conditions. These viewpoints can be used, at least conceptually, to help explain why the majority of Swiss chiropractors seem to feel that prescription privileges are an advantage for the profession, and why the minority do not. For instance, in the current study, the *prescribers* and the *integrators* favoured medication prescribing in chiropractic practice because they felt this was an important tool in the management of patients with MSK conditions. *Integrators* also believed that such privileges have allowed for better integration of chiropractors within the healthcare system. Indeed, chiropractic is one of five government-recognized medical professions in Switzerland (along with human medicine, dental medicine, veterinary medicine, and pharmacology); chiropractic services are also fully covered under the country’s publicly-funded national health insurance program [1, 7, 27]. In contrast, the *non-prescribers* were opposed to medication prescribing in chiropractic practice, feeling that chiropractic treatment should remain “drug-free” and be provided to patients as an alternative to medication. Although *collaborators* were also generally opposed to medication prescribing by themselves, this was based on the viewpoint that chiropractors needed more training in pharmacology and toxicology. Research involving other independent prescribing professionals such as nurses and pharmacists has shown that continuing education in pharmacology can improve practitioners’ confidence in prescribing medications [28], suggesting that the *collaborators* might convert to *prescribers* if given such additional training.

In terms of ChiroSuisse members’ beliefs toward the adequacy of their current pharmacology training, respondents in all four groups, at least to some extent, felt that with prescription privileges chiropractors should receive more continuing education about medications and side effects. This is consistent with the results from a study of Swiss chiropractors conducted by Wangler et al. [5], where nearly all respondents (91%) agreed that continuing education in pharmacology was a necessary component of the privilege of prescribing medications. In the current study, all four groups also either agreed (i.e., *prescribers*, *collaborators*, and *integrators*) or were at least neutral (i.e., *non-prescribers*) with the idea that a review of new medications relevant to chiropractic practice should be organized for the profession every 2–5 years. In New Mexico (USA), chiropractors complete 10 h of continuing education per year in pharmacology, toxicology, or medication administration to maintain their ‘advanced practice certification’ for prescribing medications in that state [29]. Outside of Switzerland, there have been no other published studies of chiropractors’ attitudes toward their existing medication prescription privileges. However, a majority of chiropractic patients from a 2011 survey in New Mexico [30] endorsed chiropractors’ use of limited prescription privileges when appropriately trained. In the United Kingdom, the provision of limited prescription privileges to physiotherapists and podiatrists with advanced prescribing qualifications has also been met with support from medical doctors [1, 31].

The differences identified between the four factors in the current study indicate that other contextual variables might influence chiropractors’ viewpoints about medication prescribing for MSK conditions. For instance, previous surveys of chiropractors in North America [9–11] have demonstrated that respondents who were either aligned with a focused (or ‘straight’) ideological style of chiropractic practice [9, 10], had been in clinical practice longer [10], or had graduated from an American chiropractic educational institution [11], were more likely to express opposition to chiropractors prescribing medications in clinical practice. However, in the current study, no relationships were found among the four factors and the number of years a chiropractor had been in clinical practice or the educational institution where s/he received training. As government-recognized primary contact practitioners [1, 7, 8], Swiss chiropractors are legally obliged to provide treatment that is therapeutically purposeful. As such, ChiroSuisse members were not questioned about their ideological views toward chiropractic practice because models of care offering non-essential medical services cannot be reimbursed by the Swiss compulsory health insurance (obligatorische Krankenpflegeversicherung) [27]. However, when asked about their type of practice or collaborations with other medical professionals, we found no differences between groups. In fact, the majority of respondents in all four factors indicated they collaborated with general practitioners and/or specialists on a daily basis. It is possible that Swiss chiropractors are more homogenous than chiropractors in other countries. For example, in the 2009 ‘Swiss Chiropractic Job Analysis Survey’ [7], Swiss chiropractors were shown to be more uniform than chiropractors from the United States or the United Kingdom regarding the types of conditions they treated in clinical practice, their use of diagnostic imaging, the number of continuing education hours they accrued annually, and the interprofessional relationships and referral patterns they had with medical doctors.

A concern that is often raised by those who are against chiropractors gaining access to medication prescribing rights relates to the perception that the chiropractic profession might lose its distinct (i.e., ‘non-drug’) brand and identity [1, 2]. Some warn of the experience of the osteopathic profession in the United States, where given the option of prescribing medications, the role of manipulative therapy has diminished (and nearly vanished) from that profession [1, 32]. Some *non-prescribers* also alluded to this issue in their comments provided for the current study. However, Swiss chiropractors have maintained their profession’s distinct identity despite having limited prescription privileges since 1995 [4, 7]. Swiss chiropractors are also able to choose their own individual and unique practice styles [7]. In the current study, there was strong consensus among all ChiroSuisse members that manual therapy (i.e., spinal manipulation) should not be replaced by medication prescribing in chiropractic practice. In fact, this was the only ‘consensus’ statement found among all four groups, and it contradicts the aforementioned concern that chiropractors will stop manually treating their patients if they are granted limited prescription privileges.

Using quantitative survey methods, this study queried Swiss chiropractors about the frequency with which they prescribed medications. In line with previously published research conducted in 2009 [7], 2010 [5], and 2015 [8], a total of 185 respondents reported prescribing medications infrequently in clinical practice. From the Q-methodology analysis, even the *integrators*, who had the highest reported frequency of medication prescribing among the four identified groups, still only prescribed medications to a maximum of 25% of their patients. In comparison, Swiss medical doctors prescribe medications to nearly two-thirds of patients with spine-related / MSK complaints [8]. Similar differences in rates of analgesic medication prescribing among patients receiving chiropractic services versus medical services have been reported in previous studies [33].

Regarding their existing medication prescription privileges, ChiroSuisse members were also asked about their interest in expanding the range of current medications available to prescribe for MSK or non-MSK conditions. In general, respondents were divided over being able to prescribe opioids, while most were opposed to incorporating medications into their formulary for treating non-MSK conditions. These findings are consistent with the results of several international chiropractic surveys [1, 9–12]. In the current study, several respondents from all four groups of ChiroSuisse members further commented that non-MSK conditions were outside of the chiropractic scope of practice and that more training in pharmacology would be required for Swiss chiropractors if their prescriptive scope were to include opioid analgesics.

As for the clinical indications for prescribing by Swiss chiropractors, participants in all four groups also agreed, at least to some extent, that prescribing medications were “useful in helping patients who could not sleep because of pain.” *Prescribers*, *non-prescribers*, and *integrators* further indicated that they prescribe medications to patients in “extremely acute cases where absolutely no range of motion can be achieved and pain levels are too high.” These findings are consistent with those of a previous pilot study [5] where the majority (i.e., 72 to 92%) of Swiss chiropractors agreed that prescribing medications would be useful in these situations.

Because chiropractors share primary care status with general practitioners in Switzerland [7, 8], the results of the current study may have future implications for the chiropractic profession internationally, warranting investigation. For instance, out of the four groups, *prescribers* and *non-prescribers* both agreed with, and the *collaborators* were at least neutral to, the notion that medications should be used conservatively in the management of MSK conditions. Similarly, participants in all four groups either agreed with or were neutral to the statement, “I personally take very little to no medication on a yearly basis and think we should encourage this same approach with our patients.” Although we did not specifically ask Swiss chiropractors about their attitudes and practices toward taking patients off previously prescribed MSK medications (i.e., analgesics, NSAIDs, or muscle relaxants), several respondents indicated that, with prescription privileges, they were in a position to advise patients against improper usage of these types of medications. For example, in the Q-survey, *prescribers* indicated that they only prescribe analgesic medications “when they think it would be useful, and not every time the patient asks.” In their Q-sort comments, some *non-prescribers* indicated that limited prescription privileges allow them to counsel their patients with MSK pain against overusing or over-relying on medications prescribed to treat their condition. This concept of ‘medication counselling’ was also described by Wangler et al. [5] in their pilot study of Swiss chiropractors from Bern, Switzerland. In this study, chiropractors were shown to prescribe medications at a lower frequency than requested by their patients [5]. When combined with the low rates of prescribing reported by ChiroSuisse members in the current study, these findings reiterate that Swiss chiropractors, as a whole, exercise judicious use of prescribing in clinical practice.

If practiced across the chiropractic profession globally, such a role could very well have public health implications in light of the growing opioid crisis in numerous countries around the world [34–37]. For instance, prescription medications constitute one of several evidence-based treatment tools for managing patients with MSK conditions. This added tool would allow chiropractors to function as specialists in the MSK field, in which they could select the most suitable treatment option(s), whether pharmacological or non-pharmacological, for their patients. The assumption is that with prescription privileges, chiropractors would recommend the use of non-pharmacological therapies as first-line treatments for managing MSK pain-related disorders, in agreement with current international guidelines [38, 39]. This assumption is supported in the present study by the fact that, despite having the right to do so, Swiss chiropractors reported using their prescription privileges very infrequently. However, outside of Switzerland, there is still controversy over medication prescribing within the chiropractic profession [1, 2]. As such, further research in the form of surveys, qualitative studies, mixed methods and/or Q-methodological investigations of other chiropractors’ attitudes toward gaining limited prescription privileges would be timely.

### Strengths and limitations

This study has several strengths. First, using Q-methodology, this study revealed four distinct viewpoints toward medication prescribing among Swiss chiropractors not identified in previous cross-sectional surveys [4, 5, 7]. As such, these findings provide new insights and greater understanding into the use of medication prescription privileges from the Swiss chiropractic perspective. Second, there was a high (90%) response rate from ChiroSuisse members who were recruited to develop the concourse for this study [20]. In addition, the final survey instruments (i.e., Q-sort table and demographic questionnaire) were pilot tested and validated prior to data collection [40]. Recruitment for the main part of the study was also conducted with the entire membership of Switzerland’s national chiropractic association—ChiroSuisse, reducing the potential for selection bias [40]. Moreover, there was a 100% completion rate on 89 of 91 Q-sort tables and all 185 demographic questionnaires received [40].

A limitation of this study is that just under two-thirds of all ChiroSuisse members participated, and only half of these respondents also completed the Q-sort table. Although the overall response rate (65.4%) was high compared with many previous chiropractic surveys on medication prescription rights [1, 4, 10, 11, 41, 42], this was not the case for the Q-survey. Therefore, it is possible that the current results are not applicable to all Swiss chiropractors. However, the number of Q-sort participants exceeded the minimum required sample size [18–20]. Furthermore, it is not the proportion of the participants that is important in Q-methodology, but their viewpoints [18, 19].

## Conclusions

In using Q-methodology, this study was able to demonstrate how Swiss chiropractors prioritized their viewpoints on medication prescribing for MSK conditions in chiropractic practice. With this approach, four distinct viewpoints were identified. In response to our demographic survey, participants also reported prescribing MSK medications infrequently to patients in clinical practice. These findings suggest that, even with diversity among chiropractors, limited prescribing rights can be incorporated and conservatively used within the profession. With such privileges, chiropractors internationally would have an important role to play as MSK specialists within their respective healthcare systems. If utilized judiciously, chiropractors, working alongside general practitioners, could also have a positive influence on public health in these countries. Clinicians, associations, and health policy-makers can use the results of the current study to inform the discourse on whether to extend prescribing rights to chiropractors in other jurisdictions.

## Supplementary information

**Additional file 1.** Final Q-sample used in the Q study instrument.

**Additional file 2. **Demographic comparison of Q-sort study respondents versus Q-sort non-respondents.

**Additional file 3. **Comparison of Q-sort study respondents versus all non-respondent ChiroSuisse members by gender and region of practice.

## Data Availability

The datasets used and/or analyzed during the current study are available from the corresponding author on reasonable request.

## Abbreviations

ANOVA: Analysis of variance
CE: Continuing education
GP: General practitioner
IQR: Inter-quartile range
MD: Medical doctor
MSK: Musculoskeletal
NSAIDs: Non-steroidal anti-inflammatory drugs
P: Participant
SAS: Statistical Analysis System
SD: Standard deviation
USA: United States of America

## Footnotes

1 One participant in the *prescriber* group did not complete the demographic questionnaire.

2 One participant in the *non-prescriber* group did not complete the demographic questionnaire.
