# Supplementary material for: Attitudes, beliefs, and practices among Swiss chiropractors regarding medication prescribing for musculoskeletal conditions: a national Q-methodology study
Source: Chiropr Man Therap. 2020 Oct 20;28:54. doi: 10.1186/s12998-020-00341-6 (PMC7574492; doi:10.1186/s12998-020-00341-6)
Supplement: Supplementary file 1 — Additional file 1. Final Q-sample used in the Q study instrument. [file 12998_2020_341_MOESM1_ESM.docx]

**Additional file 1** Final Q-sample used in the Q study instrument

| **Number** | **Statement ^a^** |
| --- | --- |
| 1 | I feel my chiropractic training has adequately prepared me for prescribing medications to treat MSK conditions. |
| 2 | I think adding new drug classes (opioids) to our prescription rights would be useful. |
| 3 | I think medication prescriptions are a burden because they bring added professional responsibility to the chiropractor. |
| 4 | I believe that medication prescription rights give us better credibility among our medical colleagues. |
| 5 | I think the use of medication for back pain should be discouraged and Swiss chiropractors should lead the way. |
| 6 | I do not like to prescribe because it could interfere with other medical prescriptions (i.e., double prescription). |
| 7 | The Apotheker/pharmacien (pharmacists) are for me the best people to contact with questions regarding medication. |
| 8 | I think my knowledge for prescribing medications for treating non-MSK conditions is sufficient. |
| 9 | I think that being allowed to prescribe increases our credibility among patients. |
| 10 | I think medication prescription should only be performed by general practitioners or pharmacists. |
| 11 | I feel our medication prescription privileges have allowed for better integration within the healthcare system. |
| 12 | I personally take very little to no medication on a yearly basis and think we should encourage this same approach with our patients. |
| 13 | I feel that medication prescribed by chiropractors is not appreciated by general practitioners. |
| 14 | I believe that continuous education concerning medication prescription should be mandatory. |
| 15 | I think our prescription rights in Switzerland should be open at least to level 2 analgesics (i.e., codeine, tramadol). |
| 16 | I feel that prescribing medication is useful in helping patients who cannot sleep because of pain. |
| 17 | I believe that chiropractors should get more continuing education (CE) about medications and side effects. |
| 18 | I do not manipulate patients much anymore because prescribing medications is faster and easier. |
| 19 | I think a chiropractor prescribing medications is like a medical doctor doing manipulations, let us each focus on what we do best. |
| 20 | I believe patients choose to see a chiropractor because his/her therapy is drugless. |
| 21 | I think that our current medication prescription privileges are in line with current evidence-based practice. |
| 22 | I prescribe medication in extremely acute cases where absolutely no range of motion can be achieved and pain levels are too high. |
| 23 | I think a review of new medication relevant to chiropractic practice should be organized for the profession every 2-5 years. |
| 24 | I believe that medication is a necessary component of our treatment. |
| 25 | I think medication prescription rights for chiropractors can streamline care, helping patients with MSK complaints to avoid unnecessary visits to their medical doctor. |
| 26 | I believe it should be part of the definition of Chiropractic that we assist the body in self-healing WITHOUT the use of drugs or surgery. |
| 27 | I think that ice and painkillers, which are available without a prescription, are sufficient for our needs. |
| 28 | I am concerned when prescribing medication that the patient may omit information from their medical history (e.g., Oh yes, I am taking Beta blockers, but that is none of your concern is it?...). |
| 29 | I believe our current medication prescription privileges allow for a more complete approach to managing MSK conditions. |
| 30 | I feel that a better training would definitely be necessary if we were allowed to prescribe opioids. |
| 31 | I feel that in acute cases, pain medications can be used to alleviate the increased pain (i.e., normal side-effect during the first 24-48 hours) due to the manipulation. |
| 32 | I prescribe medication only during acute and sub-acute episodes of pain, not for long-term use. |
| 33 | I think prescribing medication is a good adjunctive to our treatment in some instances to speed up recovery. |
| 34 | I think that instead of prescribing, chiropractors should collaborate with the patient’s medical doctor for the prescription of medications. |
| 35 | I feel that prescribing painkillers and NSAIDs is a useful addition to chiropractic, particularly for patients who are in severe pain. |
| 36 | I only prescribe pain medication when I think it would be useful, not every time the patient asks. |
| 37 | I think that as MSK health specialists, chiropractors should have access to a variety of treatment options including medication. |
| 38 | I believe medications should be used conservatively in regards to patient management for MSK conditions. |

*MSK* = musculoskeletal, *NSAIDs* = non-steroidal anti-inflammatory drugs

^a^ Statements are randomly listed from 1 to 38.
