# Supplementary material for: Attitudes, beliefs, and practices among Swiss chiropractors regarding medication prescribing for musculoskeletal conditions: a national Q-methodology study
Source: Chiropr Man Therap. 2020 Oct 20;28:54. doi: 10.1186/s12998-020-00341-6 (PMC7574492; doi:10.1186/s12998-020-00341-6)
Supplement: Supplementary file 2 — Additional file 2. Demographic comparison of Q-sort study respondents versus Q-sort non-respondents. [file 12998_2020_341_MOESM2_ESM.docx]

**Additional file 2** Demographic comparison of Q-sort study respondents (*n* = 89) versus Q-sort non-respondents (*n* = 98) ^a^

| Variable | Q-sort respondents  *n* (%) ^b^ | Q-sort non-respondents  *n* (%) ^b^ | P-value |
| --- | --- | --- | --- |
| Age (years): mean (*SD*) | 49.0 (11.2) | 51.3 (11.3) | 0.108 ^c^ |
| Gender   - Female - Male | 36 (41.4)  51 (58.6) | 34 (34.7)  64 (65.3) | 0.323 ^d^ |
| Chiropractic school of graduation   - Europe - Canada - USA | 9 (10.3)  25 (28.7)  53 (60.9) | 13 (13.3)  16 (16.3)  69 (70.4) | 0.080 ^e^ |
| Region of practice   - Swiss-German - Swiss-French - Swiss-Italian | 57 (65.5)  26 (29.9)  4 (4.6) | 70 (71.4)  21 (21.4)  7 (7.1) | 0.669 ^e^ |
| Years in practice: mean (*SD*) | 21.8 (10.6) | 23.1 (11.1) | 0.301 ^c^ |

*SD* = standard deviation, *USA* = United States of America

^a^ Q-sort non-respondents completed the demographic questionnaire but not the Q-sort.

^b^ Values are expressed as the number (%) unless otherwise noted.

^c^ Two-sample t-test.

^d^ Chi-square test.

^e^ Fisher exact test.
