# Supplementary material for: Attitudes, beliefs, and practices among Swiss chiropractors regarding medication prescribing for musculoskeletal conditions: a national Q-methodology study
Source: Chiropr Man Therap. 2020 Oct 20;28:54. doi: 10.1186/s12998-020-00341-6 (PMC7574492; doi:10.1186/s12998-020-00341-6)
Supplement: Supplementary file 3 — Additional file 3. Comparison of Q-sort study respondents versus all non-respondent ChiroSuisse members by gender and region of practice. [file 12998_2020_341_MOESM3_ESM.docx]

**Additional file 3** Comparison of Q-sort study respondents (*n* = 89) versus all non-respondent ChiroSuisse members (*n* = 197) by gender and region of practice

| Variable | Q-sort respondents  *n* (%) | All non-respondent ChiroSuisse members  *n* (%) ^a^ | P-value |
| --- | --- | --- | --- |
| Gender   - Female - Male | 36 (40.4)  53 (59.6) | 57 (28.9)  140 (71.1) | 0.053 ^b^ |
| Region of practice   - Swiss-German - Swiss-French - Swiss-Italian | 58 (65.2)  27 (30.3)  4 (4.5) | 129 (65.5)  60 (30.4)  8 (4.1) | 0.982 ^b^ |

^a^ Demographic data provided by ChiroSuisse. These data include all ChiroSuisse members who did not complete the Q-sort. (Note: the association does not keep demographic information on its members other than gender and region of practice [23].)

^b^ Chi-square test.
